# ER-positive endocervical adenocarcinoma mimicking endometrioid adenocarcinoma in morphology and immunohistochemical profile: A case report of application of HPV RNAscope detection

**DOI:** 10.1097/MD.0000000000024927

**Published:** 2021-04-02

**Authors:** Ruichao Chen, Ping Qin, Qiuping Luo, Wen Yang, Xuexian Tan, Tonghui Cai, Qingping Jiang, Hui Chen

**Affiliations:** Department of Pathology, the Third Affiliated Hospital, Guangzhou Medical University, Guangzhou, China.

**Keywords:** case report, HPV RNAscope, mucin-depleted, usual-type endocervical adenocarcinoma

## Abstract

**Rationale:**

Usual-type endocervical adenocarcinoma (ECA), high-risk HPV associated, is the most common type of glandular carcinoma in the endocervix. Mucin-depleted usual-type ECA is 1 end of morphological lineage of usual-type ECA and morphologically may show endometrioid features, which could cause diagnostic challenge with uterine endometrioid adenocarcinoma (EEC) and primary endometrioid ECA, especially in the setting of small biopsy and endocervical curettage (ECC).

**Patient concerns:**

A 37-year-old women presented with dyspareunia for 1 year, showing atypical glandular cell on a liquid-based Pap TCT examination and positive for HPV16 detection. ECC showed EEC in another hospital based on its “endometrioid” morphology and immunohistochemical profiles (ER/PR/PAX8 strongly positive, though p16 also strongly positive).

**Diagnoses:**

The specimen of hysterectomy in our hospital displayed a lesion confined to the uterine cervix showing the same morphology and immunohistochemical profiles as ECC. Finally, we successfully performed HPV RNAscope and detected high-risk human papilloma virus (HPV) E6/E7 mRNA particles in tumor cells in situ, which warranted usual-type ECA with mucin-depleted feature, a rare deviation of usual-type of ECA.

**Interventions:**

The patient underwent total hysterectomy with lymph node dissection.

**Outcomes:**

To date, 14 months after surgery, the patient is well without recurrence or distant metastasis, and undergoes regular reexamination.

**Lessons subsections:**

We report a rare case of mucin-depleted usual-type ECA showing overlapping morphological and immunohistochemical profiles with EEC. The pathological diagnosis was confirmed by high-risk HPV RNAscope detection which is superior than immunohistochemistry to identify usual-type ECA, warranting an important role in assisting the diagnosis of morphological vague cases.

## 1 Introduction

In the female reproductive system, endocervical cancer ranks the third behind uterine corpus and ovarian cancer globally,^[1]^ while in China, endocervical cancer ranks the first.^[2]^ With the widely application of papanicolaou smear screening and high-risk human papilloma virus (HPV) testing, the incidence and mortality rates of endocervical cancer have significantly reduced in recent years. However, this situation is limited to squamous cell carcinoma. Endocervical adenocarcinoma (ECA) is still increasing might because of scanty glandular tumor cells in the smear samples and their deceptive appearance.^[3–5]^

The usual-type ECA is the most type of all cervical adenocarcinomas, followed by gastric-type, intestinal, endometrioid, clear cell, and villoglandular carcinoma according to the 2014 WHO classification of tumors of female reproductive organs.^[6]^ The newly International Endocervical Adenocarcinoma Criteria and Classification (IECC) defined usual-type ECA as HPV related.^[7]^ Histopathological diagnosis for usual-type ECA is straightforward in most cases, which microscopically shows moderate to severe dysplasia in glands and cells accompanied by high apoptotic bodies and mitotic figures (MFs). Besides, MFs are often typically present in the upper portion of cytoplasm or “floated” in the lumen. Immunohistochemically, usual-type ECA generally strongly expresses p16 and Ki-67 with no or weak ER/PR expression.^[5–7]^ Endometrioid differentiation or manifesting endometrioid features in uterine cervix can also occur. Differential diagnoses include metastatic or primary endometrioid carcinoma and mucin-depleted usual-type ECA. These 3 entities all may show somewhat columnar cells with pseudostratified nuclei no more than moderate dysplasia and little or no mucin in cytoplasm, thus may pose diagnostic challenge in practice, especially in curettage setting. IHC may aid in this scenario. In majority, metastatic endometrial endometrioid carcinoma from corpus uteri or lower uterine segment as non-HPV related often shows patchy p16 expression, relatively lower Ki-67 proliferation index and diffuse ER/PR/PAX8 expression.^[6,8]^ On contrary, mucin-depleted usual-type ECA is more consistent with usual-type ECA. Primary endometrial ECA is extremely rare in newly IECC classification,^[7]^ which is defined as non-HPV related showing at least focal “confirmatory endometrioid features” with limited experience of immunohistochemical patterns. This impercipient type is an exclusive diagnosis after mucin-depleted usual-type ECA and metastatic endometrial endometrioid carcinoma is excluded. Here, we report a case of mucin-depleted usual-type ECA resembling endometrioid adenocarcinoma (EEC) in morphology and immunohistochemistry (IHC) and confirm the final diagnosis with the aid of high-risk HPV RNAscope detection to assure high-risk HPV infection in situ.

## 2 Case presentation

A 37-year-old women presented with dyspareunia for 1 year and went to another hospital for help. Her liquid-based Pap TCT examination prompted atypical glandular cell combined with HPV 16+. Endocervical curettage (ECC) was performed and she was diagnosed as EEC. She came to us for consultation. Histopathologically, the lesion showed a few irregular glands in confluent, cribriform, and papillary architecture, with lining pseudostratified columnar cells showing mild to moderate nuclear atypia. No mucin was in the cytoplasm. MFs and apoptotic bodies were occasionally noted but scarcely seen floating in the lumen. Immunohistochemical stains showed PAX8, ER, and PR strongly positive, while p16 was also strongly positive. Histopathological findings suggested adenocarcinoma with endometrioid differentiation except for abnormal p16 overexpression and HPV 16+. Thus, we cannot determine whether it was cervical or endometrial origin. We recommended colposcopy and fractional curettage.

### 2.1 Colposcopy and fractional curettage finding

**Figure 1:**
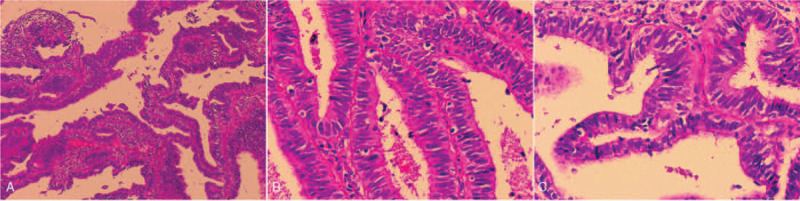
ECC of a “endometrioid” morphology. (A) The atypical glands of the tumour in biopsy specimen (H&E stain, ×40). (B) The neoplastic glands shows a pseudostratified architecture and cells are tall columnar, with eosinophilic cytoplasm and lack mucin (H&E stain, ×200). (C) Papillary-like architecture with smooth contours, small amount of apoptotic bodies and MFs present in the upper portion of cytoplasm. (H&E stain, ×200).

Colposcopy revealed cervical blood contact positive at 12 to 3 o’clock, thick vinegar white epithelium at 7 o’clock, 12 o’clock, and 1 o’clock. Fractional curettage samples were obtained. Microscopically, endometrium biopsy was negative and ECC showed the same morphological features as former consultant slide (Fig. 1). Still, we could not determine whether the adenocarcinoma originated from endocervix or lower uterine segment.

### 2.2 Hysterectomy specimen finding

Eventually, an extensive total hysterectomy with pelvic lymph node dissection was performed, but retaining bilateral ovaries.

**Figure 2:**
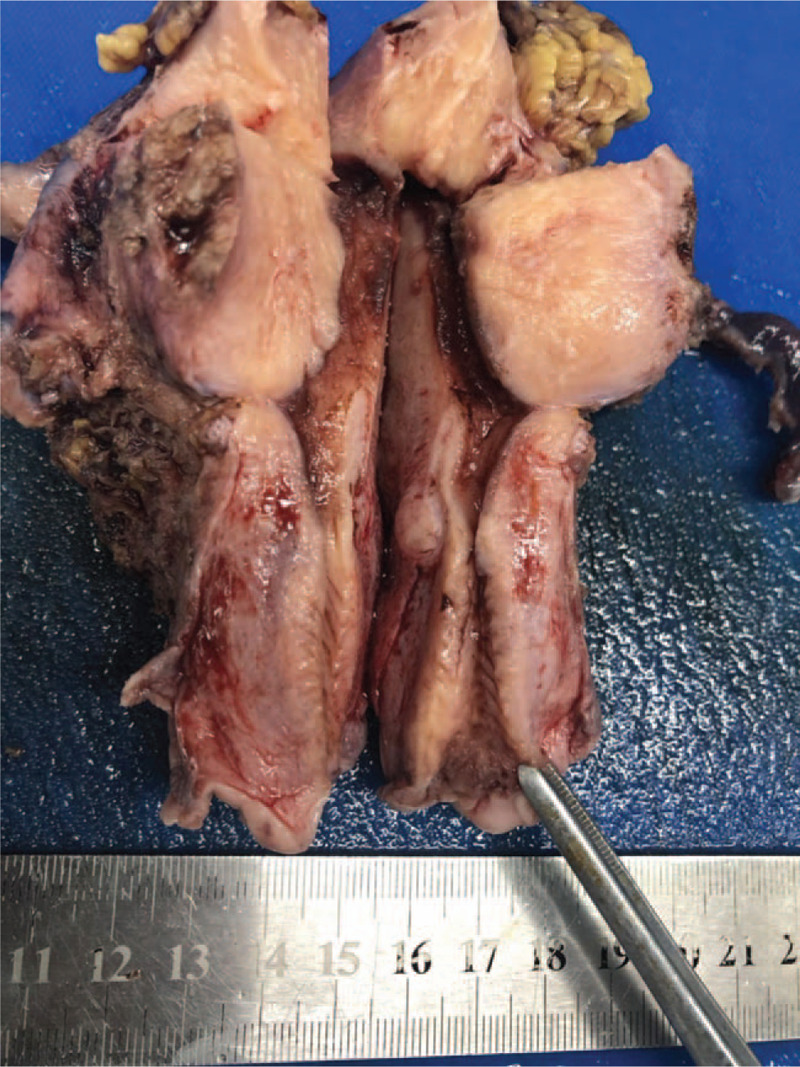
Gross view of the tumour, located in the cervix, 2 × 1 × 0.6 cm in size.

**Figure 3:**
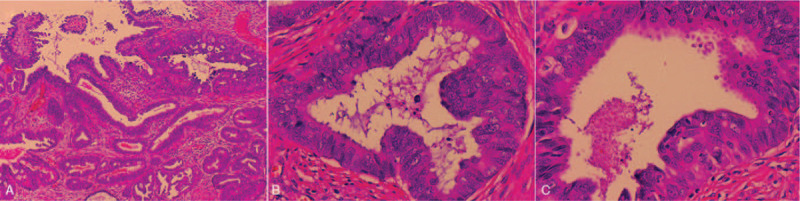
Similar to ECC biopsy specimens, crowded irregular glands infiltrate the desmoplastic stroma (A), tumor cells lack mucin and have scant, deeply eosinophilic cytoplasm mimicking the endometrial-type epithelium (B+C).

**Figure 4:**
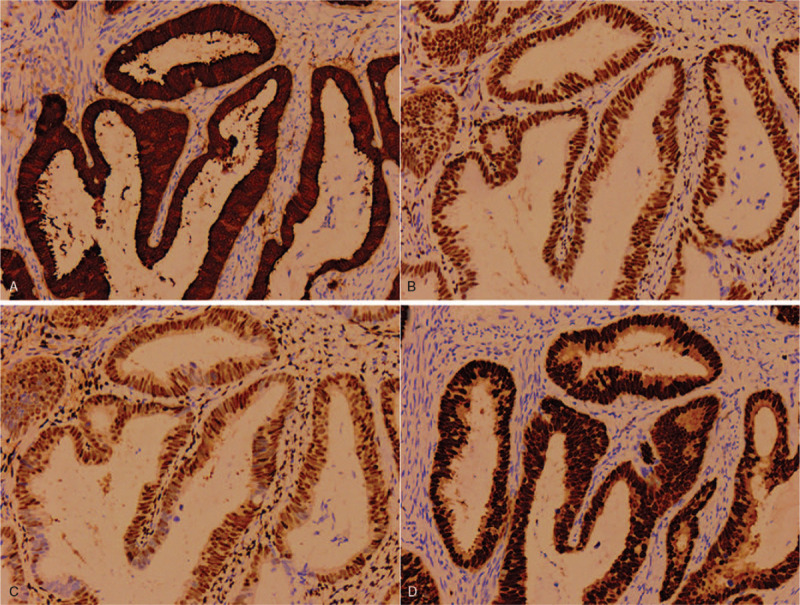
Immunostaining of the tumour, positive for P16(A), ER(B), PR(C), and PAX8(D) (×100).

**Figure 5:**
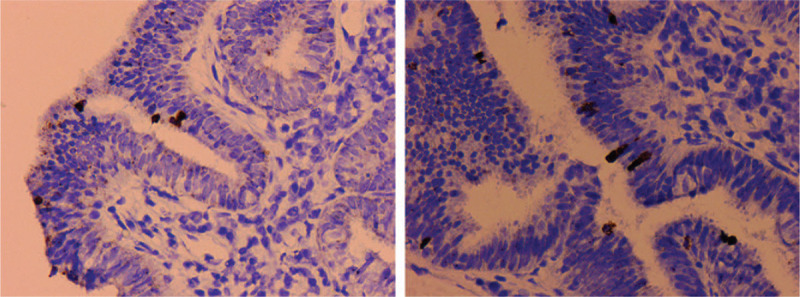
HPV-16 RNA Scope shows speckled nuclear signals (×100).

Grossly, a wide-based exophytic mass measuring 2 × 1 × 0.6 cm in size was noted at endocervical canal 1 to 5 o’clock (Fig. 2). Microscopically, the tumor consisted of irregular cystic and tubular glands with focal intraluminal papillary infoldings and cribriform architecture. The neoplastic glands showed characteristic pseudostratified tall columnar cells with eosinophilic cytoplasm and lack of mucin. The nuclei no more than moderate atypia were enlarged, elongated and perpendicular to the lumen with inconspicuous nucleoli. Apoptotic bodies and pathological MFs occasionally were seen, seldom floating in the lumen (Fig. 3). Immunohistochemically, ER, PR, and PAX8 were moderately to strongly positive in tumor cells (Fig. 4). All above features consistent with former ECC biopsy and still supported the diagnosis of EEC. However, endometrium and lower uterine segment were both negative in gross and microscopical examination. Thus, we excluded metastatic EEC arising from the uterus. In the newly IECC classification, primary endometrioid ECA is extremely rare and defined as non-HPV associated. In this case, the patient was HPV 16+ and tumor cells exhibited diffuse P16 expression with highly increased Ki-67 proliferation index which more accorded with usual-type ECA, though neither immunomarkers were 100% specific to indicate HPV infection. Nevertheless, we classified the tumor as a primary ECA, mucin-depleted usual-type, but displaying endometrioid morphology and immunohistochemical phenotypes. In order to verify our conjecture, we performed a high-risk HPV RNAScope detection to the tumor slide by RNAscope 2.0 BROWN assay kit as per the manufacturer's instructions. Briefly, 4 μm sections were hybridized with target-specific probes for the E6 and E7 mRNA of high-risk HPV genotypes after deparaffinized and pretreated with heat and protease. Ubiquitin C and the bacterial gene, DAPB, were used as positive and negative controls. Positive HPV test results was defined as punctate staining that were localized to the cytoplasm and/or nucleus of any malignant cells, and is at least 3 times stronger than DAPB staining when the staining was present in negative controls. In this case, speckled nuclear signals showed up in the nuclei indicating the presence of E6 and E7 mRNA of high-risk HPV (Fig. 5). With the reliable evidence of high-risk HPV infection, we finally confirmed the diagnosis as mucin-depleted usual-type ECA, HPV-associated, stage IB1.

The patient undergoes regular physical examination every 6 months. To date, 14 months after surgery, the patient is well without recurrence or distant metastasis.

## 3 Discussion

Accurate pathological diagnosis of ECA is crucial, especially in curettage samples. Primary usual-type ECA and metastatic EEC from corpus uteri or extended from lower uterine segment share different clinical behavior, surgical options, lymphadenectomy ranges, and postoperative treatment.^[9]^ Accurate diagnosis is important as it may affect patient's living quality and prognosis.^[9]^ Luckily, in most scenarios, these 2 entities can be identified by different morphology and IHC.^[5]^

Usual-type ECA is almost always associated with high-risk HPV, most common types are 18, 16, and 45.^[10,11]^ Nuclei of the tumor cells are always moderate to severe atypia. MFs and apoptosis can be easily identified, their presence in the upper portion of cytoplasm or “floating” in the lumen is the manifestation of high-risk HPV infection, which is a reliable diagnostic criterion to distinguish HPV and non-HPV associated adenocarcinoma.^[7]^ EEC arising from lower uterine segment is typically HPV-negative.^[8]^ Differentiating ECA from uterine EEC often requires IHC. Usual-type ECA usually shows diffuse p16 strong expression (essentially all tumor cells are positive), which is related to high-risk HPV mediated molecular changes that lead to p16 overexpression. In addition, hormone receptors expression is typically negative, but some may retain ER expression (sometimes reduced, weaker, and patchy/focal compared with the typically diffusely strong expression in normal glands), while PR is often negative, so PR is more discriminatory than ER.^[12]^ Besides, ECAs also show strong to weak nuclear PAX8 positivity, limiting its application in discrimination.^[13]^ In general, ER-/PR-/diffuse p16+ supports the diagnosis of cervical primary. On the contrary, ER+/PR+/patchy p16+ is more inclined to uterine EEC. However, rare cases may show overlapping IHC patterns, as our case, which showed strongly diffusely expressing ER/PR/P16/PAX8, which may cause diagnostic dilemma especially in ECC setting even with ER overexpression. But some high grade uterine EECs may show p16 overexpression.^[14]^ Nevertheless, our case obtained clear endometrium and lower uterine segment which confirm endocervical origin.

IECC defines usual-type ECA as 0% to 50% of tumor cells harboring appreciable intracytoplasmic mucin.^[7]^ Mucin-depleted usual-type ECA is 1 end of morphological lineage of usual-type ECA with no mucin in cytoplasm and may display mild to moderate nuclear atypia, which morphologically favors endometrioid-type differentiation. The distinction between mucin-depleted usual-type ECA and truly primary endometrioid ECA is vague according to the 2014 WHO classification. Diagnostic criteria for primary endometrioid ECA is not established in the literature. In consequence, different studies reported long range of incidence rate for endometrioid ECA from ∼7% to more than 50%.^[5,6,15]^ Most experts agreed that only tumors with scant eosinophilic cytoplasm without apparent intracytoplasmic mucin should be classified as endometrioid.^[5]^ The IECC classification criteria for endometrioid ECA are at least focal confirmatory endometrioid features and non-HPV associated. Strict to IECC criteria, only 3 cases of endometrioid ECA was identified among 409 cases of ECA.^[7]^ Two of these 3 cases strongly expressed p16 might as for their high-grade nuclei. In our case, tumor nuclei exhibited less than medium grade but p16 overexpression contradicting to IECC cases. Regardless of ER/PR expression, p16/ki-67 overexpression plus HPV 16+ clinical history in our case seems to support HPV association.

To confirm the infection of high-risk HPV, a new RNA in situ hybridization assay called RNAscope is recommended, which could be applied to routine paraffin samples for biomarker analysis. It can use a unique probe for signal amplification with no background staining while preserve tissue morphology.^[16]^ RNAscope has been used in cancer research such as breast cancer, lung cancer, Hodgkin lymphoma, ovary carcinoma, and prostate cancer.^[17]^ Compared with IHC, RNAscope can improve biomarker specificity and sensitivity that help in the detection of the extremely low biomarker expression.^[18]^ RNAscope HPV-test has also been used in the diagnosis of oropharyngeal cancers. Mirghani et al showed that RNAscope HPV-test combined with routine IHC examination is helpful in the diagnosis of HPV driven oropharyngeal cancer.^[19]^ In our RNAscope assay, the probes target 18 types of high-risk HPV E6/E7 mRNA. Positive signals are foolproof evidence of high-risk HPV infection and are more sensitive and specific than p16 IHC. As we know, gastric-type and clear cell type ECA may also show p16 overexpression, both variants are non-HPV associated.^[7,20,21]^ Our case spotted speckled nuclear signals that suggested high-risk HPV infection in the tumor cells and confirmed the diagnosis of mucin-depleted usual-type ECA.

## 4 Conclusion

In this case report, we describe a rare case of mucin-depleted usual-type ECA mimicking EEC in morphological and immunohistochemical profiles. Endometrioid features in ECA may cause diagnostic dilemma in routine pathological practice. Differential diagnosis includes metastatic EEC from uterus, primary endometrioid ECA and mucin-depleted usual-type ECA. ER/PR/P16/Ki-67 IHC panel may solve most cases. For rare cases showing overlapping IHC patterns, high-risk HPV RNAscope detection is a reliable and robust auxiliary tool in confirming high-risk HPV infection and identify mucin-depleted usual-type ECA. Primary endometrioid ECA is extremely rare and more cases are required for further understanding its clinicopathological features.

## Author contributions

**Conceptualization:** Ruichao Chen.

**Formal analysis:** Qiuping Luo, Wen Yang, Xuexian Tan.

**Immunohistochemistry and RNAscope detection:** Qiuping Luo, Wen Yang.

**Project administration:** Tonghui Cai.

**Tissue sections:** Xuexian Tan, Tonghui Cai.

**Writing – original draft:** Ruichao Chen, Ping Qin.

**Writing – review & editing:** Qingping Jiang, Hui Chen.
